# Unlocking the full potential of spatial omics in plants: practical challenges, solutions, and a path forward

**DOI:** 10.1093/plcell/koag282

**Published:** 2026-09-15

**Authors:** Min-Yao Jhu, Max Minne, Ziliang Luo, Hannah Dörpholz, Jie Yao, M Shahid Mukhtar, Fern Mathieu, Marta Peirats-Llobet, Travis Lee, Pau Formosa-Jordan, Siyu Song, Marc Libault, Che-Wei Hsu, Trevor M Nolan, Tatsuya Nobori, Christopher R Anderton, Robert J Schmitz, David Jackson, Miguel Moreno-Risueno, Hilde Nelissen, Rüdiger Simon, Rosangela Sozzani, Keiko Sugimoto

**Affiliations:** Crop Science Centre, Department of Plant Sciences, University of Cambridge, Cambridge CB3 0LE, United Kingdom; Donald Danforth Plant Science Center, St. Louis, MO 63132, United States; RIKEN Center for Sustainable Resource Science, Tsurumi, Yokohama, Kanagawa 230-0045, Japan; Department of Genetics, University of Georgia, Athens, GA 30602, United States; Institute of Bio- and Geosciences (IBG-4: Bioinformatics), CEPLAS, BioSC, Forschungszentrum Jülich, Jülich 52428, Germany; Department of Genetics, University of Georgia, Athens, GA 30602, United States; Department of Genetics and Biochemistry, Clemson University. Clemson, SC 29634, United States; University of California Davis Genome Center, University of California. Davis, CA 95616, United States; Department of Ecological, Plant and Animal Sciences, La Trobe Institute for Sustainable Agriculture and Food, School of Agriculture, Biomedical and Environmental Sciences, La Trobe University, Bundoora, Victoria 3086, Australia; Plant Biology Laboratory, The Salk Institute for Biological Studies, La Jolla, CA 92037, United States; Department of Plant Developmental Biology, Max Planck Institute for Plant Breeding Research, CEPLAS, Cologne 50829, Germany; The Sainsbury Laboratory, University of East Anglia, Norwich Research Park, Norwich NR4 7UH, United Kingdom; Division of Plant Science and Technology, Interdisciplinary Plant Group, Christopher S. Bond Life Science Center, University of Missouri, MO 65211, United States; Division of Biology and Biological Engineering, California Institute of Technology, Pasadena, CA 91125, United States; Division of Biology and Biological Engineering, California Institute of Technology, Pasadena, CA 91125, United States; The Sainsbury Laboratory, University of East Anglia, Norwich Research Park, Norwich NR4 7UH, United Kingdom; Environmental Molecular Sciences Division, Pacific Northwest National Laboratory, Richland, WA 99354, United States; Department of Genetics, University of Georgia, Athens, GA 30602, United States; Cold Spring Harbor Laboratory, Cold Spring Harbor, NY 11724, United States; Centro de Biotecnología y Genómica de Plantas (Universidad Politécnica de Madrid (UPM), Instituto, Nacional de Investigación y Tecnología Agraria y Alimentaria-CSIC (INIA-CSIC), Madrid 28223, Spain; Department of Plant Biotechnology and Bioinformatics, Ghent University, Gent 9052, Belgium; VIB Center for Plant Systems Biology, Gent 9052, Belgium; Institute of Developmental Genetics, Heinrich Heine University Düsseldorf, CEPLAS, Düsseldorf 40225, Germany; Department of Plant and Microbial Biology and NC Plant Sciences Initiative, North Carolina State University, Raleigh, NC 27695, United States; RIKEN Center for Sustainable Resource Science, Tsurumi, Yokohama, Kanagawa 230-0045, Japan; Department of Biological Sciences, Faculty of Science, The University of Tokyo, Bunkyo-ku, Tokyo 113-8654, Japan

## Abstract

Spatial omics technologies provide new opportunities for plant biology by enabling molecular profiling within structurally intact tissues, revealing spatially organized cell states, developmental gradients, and regulatory interactions. While spatial transcriptomics has driven early advances, the field is rapidly expanding toward integrated spatial multi-omics by combining single-cell and spatial transcriptomic, epigenomic, proteomic, and metabolomic data. These approaches offer new opportunities to study development, physiology, and plant biotic and abiotic interactions in spatially preserved cellular contexts. However, despite rapid adoption, the field remains constrained by plant-specific challenges when applying technologies largely developed for animal systems. Compared with animal systems, plant tissues pose additional challenges due to rigid cell walls and diverse chemistries, complicating sample preparation, cell and subcellular segmentation, signal detection, and data integration. As a result, many studies rely on bespoke protocols and analysis pipelines that are often difficult to reproduce or generalize. Here, we provide a practical, solution-oriented synthesis of current bottlenecks across experimental and computational pipelines, highlight emerging strategies to overcome these limitations, and propose a roadmap for community-driven protocol sharing, benchmarking, and integration across spatial and multi-omics modalities. Addressing these challenges will be essential to establish spatial omics as a routine and scalable tool for plant biology.

## Introduction

Plant development, cell identity, signaling, and metabolism are tightly linked to tissue organization. This spatial organization underpins key processes, including pattern formation, long-range signaling, and localized responses to developmental and environmental cues. Omics approaches have provided large amounts of data to understand these key processes and combined with dissociation-based single-cell methods; we are much closer to understanding their cellular basis. However, a dissociation-based single-cell method disrupts positional context. Spatial omics addresses this gap by enabling molecular profiling directly within structurally intact tissues, preserving both cellular identity and anatomical relationships.

Spatial transcriptomics (ST) (Marx 2021; Moses and Pachter 2022) has become the primary entry point for spatial omics in plants (Duncan et al. 2016; Giacomello et al. 2017). While earlier approaches, including antibody-based spatial proteomics and mass spectrometry (MS) imaging, enabled spatially resolved molecular measurements, ST has rapidly expanded our ability to resolve gene expression patterns across tissues, organs, and developmental stages and enhanced our understanding of spatially organized transcriptional programs, cellular heterogeneity, and local signaling environments. The field is now progressing toward integrated spatial multi-omics through the combination of ST with single-cell transcriptomic, chromatin accessibility, spatial proteomic (SP), and spatial metabolomic (SM) approaches (Nobori 2025; Nobori et al. 2025; Zhang et al. 2025; Qin and Xia 2026; Shi et al. 2026; Tao et al. 2026; Yao et al. 2026).

Here, we argue that one key barrier to progress is no longer technology availability but the lack of plant-adapted, standardized, and benchmarked workflows spanning the full spatial omics pipeline, from sample preparation to data interpretation. Building on existing conceptual reviews, we focus on identifying where current workflows fail in practice, how these limitations affect biological interpretation, and which emerging strategies provide realistic paths forward. Together, we provide a solution-oriented, plant-specific discussion of current challenges, emerging best practices, and community-level actions needed to unlock the full potential of spatial omics in plants.

## Building on the landscape: key advances and remaining gaps

ST has been successfully applied across a growing range of plant species and organs, leading to the first generation of plant spatial atlases in systems such as *Arabidopsis*, soybean, maize, rice, barley, and *Medicago* (Duncan et al. 2016; Giacomello et al. 2017; Giacomello and Lundeberg 2018; Liu et al. 2023a; Peirats-Llobet et al. 2023, 2026; Cervantes-Pérez et al. 2024; Serrano et al. 2024; Wang et al. 2024; Yao et al. 2024; Jhu et al. 2026a; Lee et al. 2025a; Tennant et al. 2025; Demesa-Arevalo et al. 2026). These datasets have revealed spatially structured cell states, developmental gradients, and tissue-specific regulatory programs that were previously obscured in bulk or dissociated single-cell analyses.

Integration of ST with single-cell or single-nucleus RNA sequencing has proven particularly powerful for resolving cell identities and developmental trajectories (Cervantes-Pérez et al. 2024; Fan et al. 2025; Nobori 2025; Zhang et al. 2025; Demesa-Arevalo et al. 2026; Qin and Xia 2026; Yao et al. 2026), but discrepancies in resolution, transcript capture or detection efficiency, and sampling depth between modalities remain a major source of uncertainty.

Emerging 3D, multimodal, and data-integration strategies extend plant spatial omics beyond 2D cell landscapes by capturing tissue architecture and cellular neighborhoods more accurately, thereby providing a richer context for interpreting spatial patterns and developmental trajectories (Mo et al. 2024; Schott et al. 2024; Peirats-Llobet et al. 2026). Combining ST with genome-wide association studies (GWAS) offers a framework to map trait-associated loci onto spatial expression patterns (Song et al. 2025; Tang 2025), providing insight into where and when candidate genes act and creating new opportunities to link genetic variation in complex traits to spatially resolved molecular phenotypes.

However, most studies remain limited to a specific context. Experimental success requires tailored protocol optimization, platform-specific adjustments, and custom computational pipelines, which are rarely transferable across tissues, species, or laboratories. As a result, spatial omics datasets in plants are difficult to compare, benchmark, and integrate at scale. Addressing this gap requires a shift toward robust, standardized workflows that can be applied reproducibly across biological contexts. In the following sections, we examine the key challenges across the spatial omics pipeline and highlight emerging strategies to overcome them.

## Current practical challenges: what is still missing

### Protocol optimization across diverse plant tissues

Sample preparation remains the single most critical determinant of success in spatial omics experiments. Although optimization is a general challenge across systems, plant tissues combine several features that amplify technical constraints, including rigid and impermeable cell walls, high water content, extensive vacuolation, abundant secondary metabolites, strong autofluorescence, and heterogeneous tissue architectures (Fig. 1) (Giacomello and Lundeberg 2018). Immature tissues with relatively regular cellular organization are generally more amenable to spatial profiling than structurally complex organs, with section orientation, developmental stage, and environmental history further influencing outcomes. Consequently, spatial omics protocols in plants require extensive optimization across organs, developmental stages, and species.

**Figure 1:**
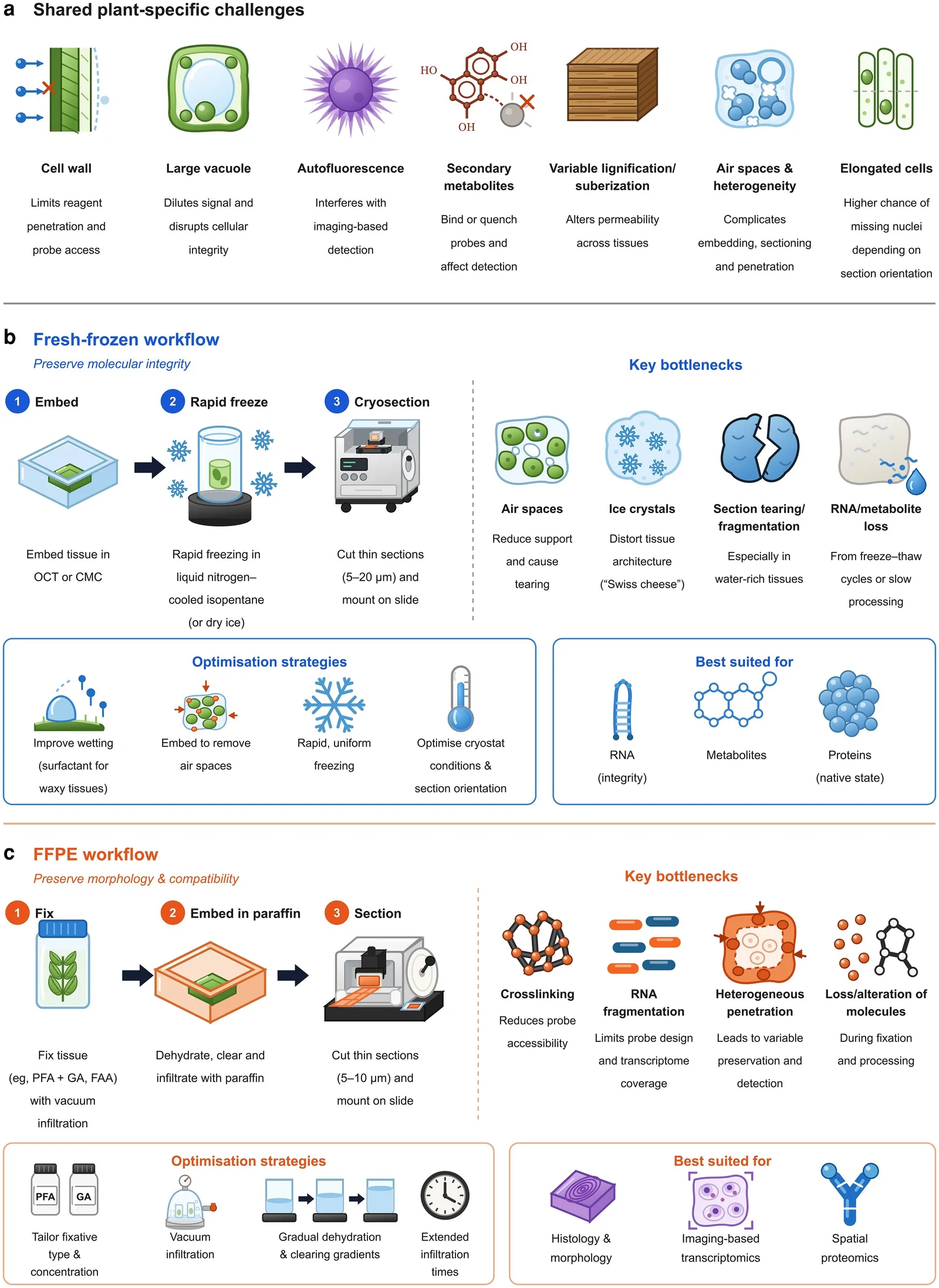
Plant tissue preparation workflows and major bottlenecks in spatial omics. Plant spatial omics workflows require extensive optimization to balance molecular preservation, tissue integrity, and compatibility with downstream analyses. a) Shared plant-specific constraints, including rigid cell walls, large vacuoles, autofluorescence, secondary metabolites, variable lignification or suberization, tissue heterogeneity and air spaces, and elongated cell morphology, can influence reagent penetration, probe accessibility, sectioning quality, and signal detection. b) Fresh-frozen workflows preserve RNA, metabolite, and protein integrity but are highly sensitive to embedding quality, rapid freezing conditions, and cryosectioning artifacts such as ice-crystal formation, tissue tearing, and molecular loss. Tissues with hydrophobic or air-filled structures may require surfactant treatment (eg Tween-20 washing) (Chaulagain et al. 2019), embedding-medium infiltration, or tissue trimming to improve cryosectioning stability and tissue support during sectioning. Rapid freezing methods, including liquid nitrogen–cooled isopentane baths or powdered dry ice workflows, are commonly used to minimize ice-crystal formation during cryoembedding. c) Formalin-fixed paraffin-embedded (FFPE) workflows provide improved tissue morphology, structural preservation, and compatibility with histological and imaging-based applications but introduce fixation-induced crosslinking, RNA fragmentation, and heterogeneous reagent penetration. Shared plant-specific constraints, including rigid cell walls, large vacuoles, autofluorescence, secondary metabolites, lignification, tissue heterogeneity, and elongated cell morphology, further influence fixation efficiency, probe accessibility, sectioning quality, and signal detection. Representative optimization strategies include rapid tissue harvesting, minimization of post-dissection delays, controlled dehydration, and avoidance of freeze–thaw cycles to preserve spatial integrity and biomolecular stability. Additional FFPE optimization strategies may include paraformaldehyde combined with low concentrations of glutaraldehyde to improve structural preservation in complex tissues (Jhu et al. 2026a), formalin–acetic acid–ethanol (FAA)-based fixation to enhance penetration in difficult plant samples, vacuum-assisted infiltration, extended solvent exchange gradients, and tissue-specific infiltration workflows adapted from classical plant in situ hybridization protocols (Jackson 1992; Jones and Howat 2020). The schematic summarizes workflow-specific technical considerations required for successful spatial omics analyses in plant tissues.

While previous reviews acknowledge these challenges, practical solutions are rarely documented in sufficient detail. Many successful studies rely on custom adaptations, such as modified fixation chemistries, tissue-specific permeabilization strategies, or tailored probe designs. This lack of standardization represents a bottleneck for reproducibility and broader adoption, highlighting the need for community-driven, plant-specific protocol repositories, ideally organized by tissue type and spatial omics platform and maintained in a versioned, updateable format.

#### Sample preparation and biological constraints on detectability

Across spatial omic platforms, sample preparation must balance preservation of the integrity and spatial distribution of biomolecules (Fig. 1). Fresh-frozen tissues typically provide higher RNA, metabolite, and protein integrity, making them well-suited for most spatial omics applications in plants. However, they require rapid processing to minimize degradation and are particularly susceptible to mechanical damage. In contrast, fixed tissues provide improved structural preservation and compatibility with longer storage and complex embedding protocols and are generally compatible with antibody-based SP approaches (Veličković et al. 2022; Wang et al. 2026a). On the other hand, fixation risks altering target transcript accessibility, washing away metabolites, masking protein epitopes, inducing crosslinking artefacts, or distorting subcellular organization due to differential penetration of fixatives through rigid cell walls and vascular tissues (Myers et al. 2025; Li et al. 2025a). Overall, fixation strategies must therefore balance rapid immobilization of biomolecules while minimizing molecular leaching, as insufficient fixation risks molecular degradation and loss of spatial fidelity, whereas over-fixation can compromise both morphology and molecular detection.

#### Fresh tissue embedding challenges and optimization

A critical step for cryosectioning is the freezing of tissues in embedding medium (eg Optimal Cutting Temperature [OCT] compound or carboxymethyl cellulose) (Dong et al. 2016), and it is essential that tissues are completely immersed. A limitation of OCT is that it can mask metabolite and proteomics signals in MS-based assays (Eriksson et al. 2013; Zemaitis et al. 2026). Spaces between organ layers (eg leaf primordia at the SAM), internal air spaces (eg in seed coat structure), irregular structures (eg trichomes), or air bubbles created during embedding reduce uniform mechanical support during sectioning and can easily lead to tearing or damage of the sections (Fig. 1). Irregular tissue architecture and internal air spaces often require tissue-specific embedding optimization to improve sectioning stability (Luo and Schmitz 2025). Maintaining rapid and uniform freezing conditions is essential for fresh tissue blocks, as slow freezing, particularly in tissues with high water content, can lead to ice crystal formation that distorts tissue architecture (Wahid et al. 2024).

SM workflows introduce additional constraints not encountered in ST. Workflows based on matrix-assisted laser desorption/ionization MS (MALDI-MS) require the application of a chemical matrix to the tissue surface to enable ionization of metabolites. Matrix deposition must be carefully controlled, as solvent exposure can lead to delocalization of small molecules. Furthermore, matrix application and subsequent removal may introduce incompatibilities with downstream transcriptomics analyses, complicating the design of workflows that aim to integrate metabolomic and transcriptomic data from the same tissue section (Kriel et al. 2026).

#### Paraffin-embedded tissue processing challenges and optimization

Formalin-fixed paraffin-embedded (FFPE) tissues represent an increasingly important alternative for spatial omics (Bai et al. 2024; Zhao et al. 2025), particularly when long-term storage, sample transport, or histological workflows are required. FFPE processing provides superior preservation of tissue morphology and structural integrity and is especially advantageous for plant tissues that are challenging to section using cryosectioning methods, including structurally heterogeneous, lignified, or suberized plant tissues. FFPE-embedded samples additionally facilitate serial ribbon sectioning, providing greater control over section position and orientation—for example, when identifying medial sections through shoot and root apical meristems or reconstructing larger structures in 3D. This can also improve consistency in spatial omics analyses because nuclei may occupy only a small fraction of the cellular volume such that section orientation and thickness influence nuclear capture, transcript detection, segmentation accuracy, and the interpretation of spatial organization.

However, fixation-induced crosslinking and RNA fragmentation impose constraints on transcript accessibility, and the embedding procedure can remove proteins or metabolites of interest or crosslink metabolites that remain in place. Consequently, FFPE-compatible ST approaches typically rely on multiple short probe sequences targeting predefined regions, which can limit transcriptome coverage and sensitivity. Emerging approaches such as in situ polyadenylation may help recover fragmented RNAs from FFPE tissues (Bai et al. 2024), although their utility in plants remains largely unexplored. Cuticle- and wax-rich shoot tissues can be more difficult to fix and permeabilize than roots because of reduced reagent penetration, while dense, lignified, or hydrophobic tissues frequently require modified fixation chemistries, vacuum-assisted infiltration, and extended solvent exchange steps to improve reagent penetration and preserve spatial integrity (Jackson 1992; Jhu et al. 2026a). Optimization of fixation, dehydration, and paraffin infiltration is therefore critical for balancing structural preservation with molecular accessibility in plant tissues (Fig. 1).

Recent studies further illustrate the importance of tissue-specific protocol adaptation. For example, workflows that utilize the Xenium platform (Fig. 2) developed for *Medicago* nodules required optimization of fixation chemistry, autofluorescence quenching, and probe design strategies (Janesick et al. 2023; Jhu et al. 2026a). In probe-based ST workflows (Groiss et al. 2021), repeated rounds of hybridization and washing can cause tissue detachment, which can be mitigated by heating mounted sections to improve tissue adhesion (Laureyns et al. 2022).

**Figure 2:**
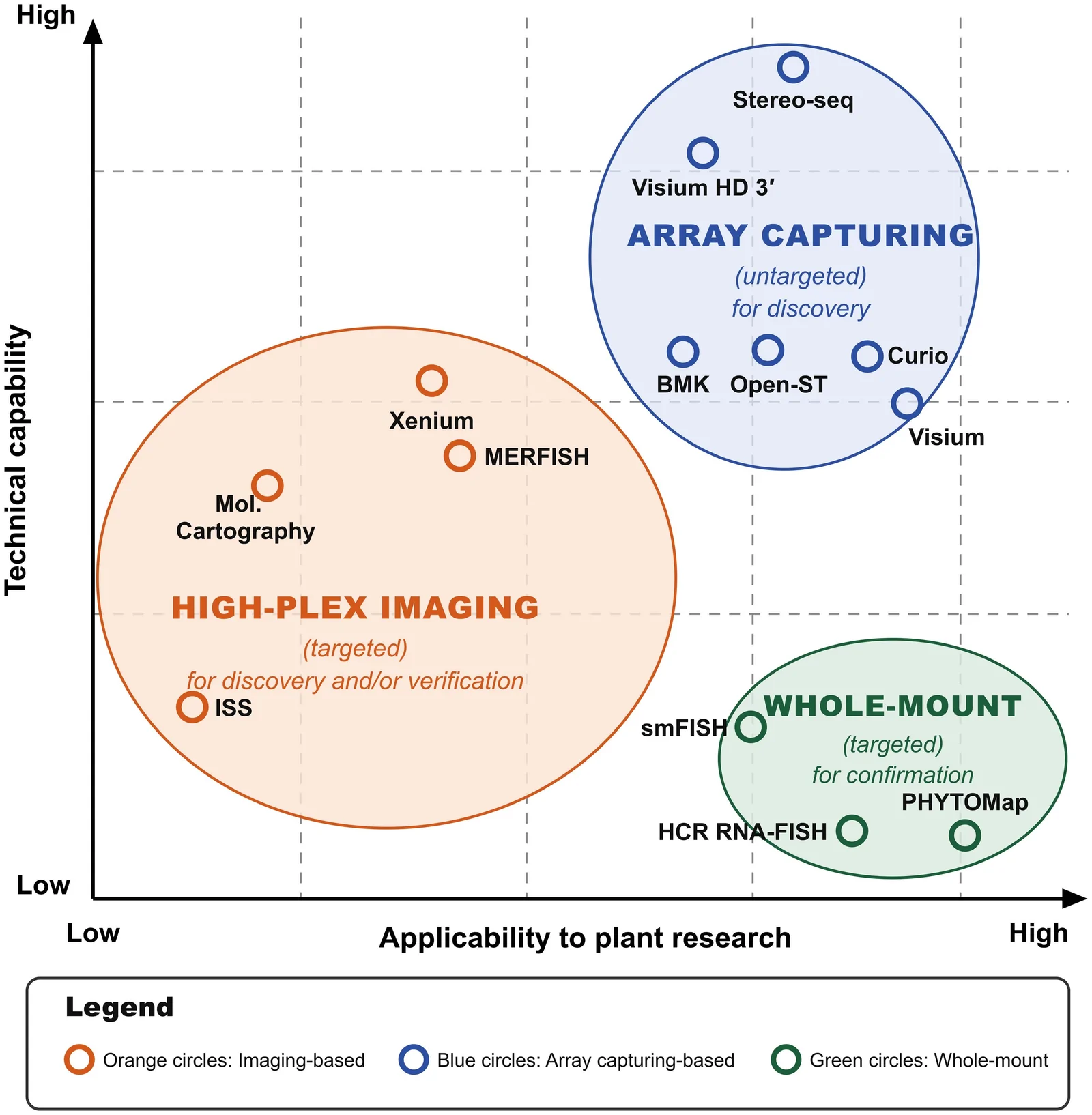
Conceptual comparison of ST platform families for plant research. ST approaches used in plant systems can be broadly grouped into 3 platform classes: array capturing-based methods, high-plex imaging-based methods, and low-multiplex whole-mount imaging approaches. These technologies occupy distinct performance regimes defined by trade-offs among transcriptome breadth, spatial resolution, throughput, and compatibility with plant tissues. Array capturing platforms, including Visium v1, Visium HD 3′, BMK (Li et al. 2025b; Wang et al. 2025), Curio (Iimura et al. 2025; Lee et al. 2025a), and Stereo-seq, provide untargeted transcriptome-wide profiling and are particularly suited for discovery-oriented applications. High-plex imaging methods such as Xenium, MERFISH (Multiplexed Error-Robust Fluorescence *In Situ* Hybridization), Molecular Cartography (Xu et al. 2025; Zhu et al. 2025), and ISS (*In Situ* Sequencing) offer targeted spatial profiling with increased spatial precision and are commonly applied for discovery and/or mechanistic validation of predefined gene sets. Whole-mount imaging approaches, including smFISH (single-molecule Fluorescence *In Situ* Hybridization), HCR RNA-FISH (Hybridization Chain Reaction RNA Fluorescence *In Situ* Hybridization), and PHYTOMap, prioritize quantitative validation and tissue-context preservation in intact plant samples. Conceptual qualitative comparisons shown across the diagram are intended to illustrate general relative strengths, limitations, and current use cases in plant spatial biology.

ST technologies can broadly be divided into 2 categories: imaging- or sequencing-based, each with distinct advantages and challenges, as discussed below.

### Spatial omics platform selection and trade-offs

Platform choice involves trade-offs among transcriptome coverage, spatial resolution, throughput, scalability, and tissue compatibility (Fig. 2). Sequencing-based approaches prioritize transcriptome breadth and discovery potential, whereas imaging-based approaches provide higher spatial precision and sensitivity for predefined targets.

#### NGS-based ST methods

The primary advantage of sequencing-based ST approaches is unbiased transcriptome-wide capture without predefined gene panels, making these platforms particularly useful for discovery and atlas construction in plant systems where comprehensive cell-type references remain incomplete (Fig. 2). Typical datasets detect tens of thousands of genes overall and hundreds to thousands per cell-sized bin, supporting comparative profiling across organs, developmental gradients, and environmental conditions (Xia et al. 2022).

Next-generation sequencing (NGS)–based ST technologies mostly use poly(dT) oligonucleotides to capture the transcriptome without bias. Current commercial platforms vary substantially in spatial resolution and cost (Table 1). High-density platforms such as Stereo-seq use DNA nanoball arrays to achieve subcellular spacing between capture spots (Wang et al 2024). However, because individual capture spots often contain limited transcript counts, neighboring spots are typically computationally grouped (“binned”) together to improve signal detection, which may decrease effective analytical resolution for small cells such as those in the root stem cell niche. Customizable ST platforms based on Illumina sequencing flow cells have also been developed (Cho et al. 2021; Poovathingal et al. 2024; Schott et al. 2024; Kim et al. 2025). For example, Open-ST (Schott et al. 2024) achieves low-cost, high-spatial resolution (Table 1) and high scalability with customizable capture-area sizes but requires substantial initial investment and technical expertise.

**Table 1:** Comparison of major plant-compatible next-generation sequencing (NGS)-based spatial transcriptomics platforms.

Platform	Physical spatial resolution	Sample compatibility	Representative plant applications	Major advantages	Major limitations
**10× Genomics Visium v1**	55 µm	FF & FFPE	Barley grain (Peirats-Llobet et al. 2023, 2026); orchid flowers (Liu et al. 2022); poplar stem (Du et al. 2023; Li et al. 2023); tomato callus (Song et al. 2023) ; maize kernel (Fu et al. 2023); *Arabidopsis* leaves (Saarenpää et al. 2024) ; Portulaca leaf (Moreno-Villena et al. 2022)	Commercial workflow; whole transcriptome; widely adopted	Low spatial resolution; mixed-cell capture; expensive
**Curio Seeker**	∼10 µm	FF	Germinating *Arabidopsis thaliana* seeds (Lee et al. 2025a)	High spatial resolution; commercial workflow; whole transcriptome	Less established ecosystem; lower transcript capture per spot; expensive
**10× Genomics Visium HD 3′**	2 µm	FF	Soybean seed and Arabidopsis seedling (10× Genomics 2026)	High spatial resolution; commercial workflow; whole transcriptome	Lower transcript capture per spot; expensive
**Open-ST**	∼0.6 µm	FF	No published plant application to date	Low cost; scalable; customizable capture areas; open source	Requires custom fabrication and optimisation; lower transcript capture per spot
**Stereo-seq**	∼500 nm	FF	*Arabidopsis thaliana* leaves (Xia et al. 2022); maize ear (Wang et al. 2024); soybean root, nodule, shoot apex, leaf, and stem (Fan et al. 2025); *Lotus japonicus* nodule (Ye et al. 2024)	Higher spatial resolution; commercial workflow; whole transcriptome	Lower transcript capture per spot; expensive

FF, fresh-frozen; FFPE, formalin-fixed paraffin-embedded.

Limitations of NGS-based ST are structural rather than merely technical. Even when bin sizes approach cellular dimensions, transcript diffusion and aggregation imply the effective measurement unit remains a spatial neighborhood rather than a single cell. This creates a mismatch with downstream analyses such as cell-type deconvolution (Gaspard-Boulinc et al. 2025) or cell–cell communication inference, which assume discrete cellular units. In plant tissues, this gap is further amplified by reliance on segmentation methods developed for animal systems that fail to account for large vacuoles, non-uniform cell shapes, and distinct nuclear morphologies, making many analyses described as “single-cell resolution” composites of multiple cells in practice (Gaspard-Boulinc et al. 2025).

Despite these challenges, NGS-based ST platforms are currently well suited for unbiased discovery and whole-transcriptome atlas construction in plants, particularly when broad transcriptome coverage across larger tissue domains is prioritized. Studies requiring precise assignment of transcripts to individual cells, particularly small or densely packed cell types, may instead benefit from complementary imaging-based approaches.

#### Imaging-based ST methods

Imaging-based ST technologies resemble traditional in situ hybridization methods, where antisense probes are hybridized to unique transcripts of interest. These methods differ in target throughput, since ST enables the simultaneous investigation of hundreds to thousands of different transcript targets (Table 2). Current imaging-based ST techniques use combinatorial barcoding of probe sets, which are visualized over multiple sequential rounds of secondary probe hybridization, imaging of the fluorescent signal, and probe stripping. These multiple imaging rounds, which often require further amplification steps to increase sensitivity, result in the generation of a spatially resolved fluorescence “barcode” that corresponds to the detection of an individual mRNA target molecule.

**Table 2:** Comparison of major imaging-based spatial transcriptomics platforms compatible with plant tissues.

Platform	Physical spatial resolution	Sample compatibility	Representative plant applications	Major advantages	Major limitations
**Molecular Cartography**	Subcellular (∼300 nm)	FFPE & FF	Rice roots (Zhu et al. 2025; Chancellor et al. 2026); maize and *Arabidopsis* inflorescence/shoot stem cells (Xu et al. 2025); Grass roots (Guillotin et al. 2023)	Medium multiplexing (custom up to 100); commercial platform	Expensive; limited gene number; targeted approach
**Hybridization-based in situ sequencing (HybISS); and fluorescence in situ hybridization (FISH)**	Subcellular (∼300 nm)	FFPE & FF, Whole mount	*Arabidopsis* roots (PHYTOMap) (Nobori et al. 2023); *Arabidopsis* root meristem (smFISH) (Zhao et al. 2023); *Arabidopsis* inflorescences and maize and sorghum roots (HCR RNA-FISH) (Huang et al. 2023b)	Flexible targeted detection from low-plex (4) to medium plex (up to ∼120)	Limited gene number; targeted approach
**10× Genomics Xenium**	Subcellular (∼200 nm)	FFPE & FF	*Medicago truncatula* roots and nodules (Jhu et al. 2026a, 2026b)	High multiplexing (pre-designed: 5000, custom up to 480); commercial platform	Expensive; targeted approach
**MERFISH**	Subcellular (∼150 nm)	FFPE & FF	*Arabidopsis thaliana* leaves (Nobori et al. 2025); seedlings, shoot apex/apical hook, stems, flowers and siliques (Lee et al. 2025a)	High multiplexing (pre-designed: 815; custom up to 960); commercial platform	Expensive; complex imaging workflows; targeted approach

FF, fresh-frozen; FFPE, formalin-fixed paraffin-embedded.

Imaging-based in situ readouts offer an advantage over ex situ sequencing-based ST approaches by directly localizing individual transcript molecules within intact tissues rather than within predefined spatial bins or spots. Depending on the platform, these methods can achieve single-molecule detection with subcellular spatial resolution, enabling the detection of low-abundance genes such as transcription factors and signaling molecules (Table 2). This high spatial precision is particularly valuable in plant systems where biological interpretation depends on localized gene expression patterns, including stomatal patterning, meristem organization, symbiotic interface formation, and pathogen responses at cellular resolution. Recent plant-compatible imaging-based workflows are addressing major technical barriers in plant tissues, including cell-wall autofluorescence and probe specificity required to distinguish paralogs and detect orthologs across ecotypes (Jhu et al. 2026a).

While imaging-based spatial methods, such as Xenium, MERFISH, Molecular Cartography, and ISS using either padlock probes with rolling circle amplification or cyclic fluorescence hybridization offer clear advantages (Janesick et al. 2023), they also present specific challenges (Table 2). Targeted approaches require a priori knowledge for probe panel design, relying on sufficient genome information and high-resolution or single-cell reference datasets to select targets of interest (Jhu et al. 2026a). In addition, several probes are required for each RNA target, and small RNAs (or highly fragmented RNAs) may therefore remain undetectable. Furthermore, signal amplification protocols involve the generation of complex probe “sandwiches”, resulting in steric hindrance and reduced detection sensitivity in very RNA-rich tissues. Whole-cell transcript quantitation and downstream single-cell analysis require high-quality cell segmentation and imputation for analysis of gene targets not present in the experiment, as discussed further in this review.

Although multiplexing capacity is rapidly increasing, and recent developments such as the 10× Genomics Atera platform suggest that whole-transcriptome imaging may become feasible in human tissues, comparable whole-transcriptome imaging-based ST approaches are not yet available for plant systems. Consequently, panel design strongly influences the biological insights that can be obtained, particularly when limited gene sets are used. Designing a probe panel requires predefined cell-type markers, creating a circular dependency in which the cell types one aims to investigate must already be characterized, making high-plex imaging studies typically downstream of scRNA-seq rather than independent discovery tools. Despite these limitations, imaging-based ST approaches provide unmatched spatial precision and sensitivity for targeted gene detection.

Low-multiplex whole-mount imaging approaches such as smFISH, HCR RNA-FISH, and PHYTOMap occupy a complementary niche within plant spatial biology by prioritizing quantitative accuracy, intact tissue context, and orthogonal validation over transcriptome breadth. These methods are particularly valuable for orthogonal validation and resolving selected targets in 3D tissue context (Fig. 2), as discussed further in Section 3.7.

Taken together, these platforms address complementary experimental needs rather than representing a simple hierarchy of performance. NGS-based ST is well suited for unbiased transcriptome-wide discovery and atlas construction, whereas high-plex imaging-based ST prioritizes cellular and subcellular localization of predefined targets. Low-plex whole-mount approaches sacrifice transcriptome breadth but provide targeted validation and 3D tissue context. Platform selection should therefore be guided by the biological question, tissue architecture, availability of prior molecular information, required spatial resolution, and whether transcriptome breadth, cellular localization, or volumetric context is the primary objective. In many studies, combining these approaches may provide a more effective strategy than relying on a single platform.

#### Beyond transcriptomics: spatial epigenomics, metabolomics, and proteomics

Although spatial transcriptomics has driven the rapid development of plant spatial omics, complementary technologies are emerging that capture additional layers of molecular regulation. Spatial epigenomics, proteomics, and metabolomics provide distinct but complementary information, ranging from chromatin state and regulatory potential to protein abundance and metabolic activity. Integrating these modalities promises a more comprehensive understanding of plant development, physiology, and responses to environmental stimuli.

Emerging spatial epigenomic technologies extend spatial transcriptomics by directly measuring regulatory states that precede transcriptional activity. Recent studies in mammalian systems have demonstrated spatial profiling of chromatin accessibility (spatial ATAC-seq) (Deng et al. 2022b; Llorens-Bobadilla et al. 2023; Farzad et al. 2024; Huang et al. 2025), histone modifications (Spatial-CUT&Tag) (Deng et al. 2022a; Farzad et al. 2024), and joint spatial epigenome–transcriptome profiling within intact tissues. Most spatial epigenomic approaches rely on microfluidic barcoding or specific barcode hybridization to capture the tagged DNA fragments. More recently, Huang et al. (2025) introduced a strategy in which DNA fragments are tagged with poly(A) tails, enabling simultaneous profiling of chromatin accessibility and transcription. Because this approach is compatible with conventional spatial transcriptomics slides containing poly(dT) capture oligos, it may also be relatively straightforward to test and adapt for plant tissues. While these approaches have not yet been widely applied in plants, they highlight the potential to reveal how chromatin accessibility and epigenetic regulation shape spatial patterns of gene expression. In the meantime, integrating plant single-cell ATAC-seq datasets with single-cell and spatial transcriptomic atlases (Nobori 2025; Nobori et al. 2025) provides a practical strategy for inferring spatial regulatory programs until plant-compatible spatial epigenomic technologies become available.

In contrast to ST, which primarily captures gene expression as a proxy for cellular state, SM and SP directly measure downstream biochemical and functional outputs. However, their application in plants presents additional challenges. Compared with animal systems, plants lack extensive antibody libraries, limiting SP approaches. SM offers rich functional insight but often at coarser spatial resolution and with challenges in molecular identification. Transparent, plant-specific comparisons between platforms are therefore essential for informed experimental design.

SM in plants is mostly performed using MSI techniques, particularly matrix-assisted laser desorption/ionization mass spectrometry imaging (MALDI-MSI). MALDI-MSI enables label-free detection of a broad range of metabolites, including primary metabolites, lipids, and specialized secondary compounds, making it particularly valuable for studying plant-specific metabolic pathways such as defense, signaling, and specialized metabolism (Huang et al. 2023a; Liu et al. 2026c). Spatial resolution typically ranges from ∼5 to 50 µm depending on instrumentation, sample preparation, and acquisition settings, although effective resolution is often limited by analyte diffusion and matrix application.

Alternative MSI approaches offer complementary trade-offs. Secondary ion MS can achieve submicron spatial resolution, enabling subcellular mapping, but is generally limited to measuring elemental composition and fragments of larger molecular species (Jia et al. 2026). Desorption electrospray ionization provides ambient ionization with minimal sample preparation, but typically at lower spatial resolution (∼10 µm) and with reduced sensitivity for certain compound classes (Colwell et al. 2026). Laser ablation atmospheric pressure photoionization MSI enables analyses at different plant organ depths, spatially resolving tissues in the Z axis, although resolution decreases to 50 µm (Hieta et al. 2021). Across all MSI platforms, key limitations include ion suppression effects, variability in ionization efficiency, and challenges in confident metabolite identification, particularly in untargeted analyses. Despite these constraints, SM is uniquely suited for mapping biochemical gradients, metabolite compartmentalization, and tissue-specific metabolic activity.

SP approaches are more heterogeneous and depend strongly on the underlying detection strategy. Antibody-based imaging methods, including multiplex immunofluorescence and imaging mass cytometry, offer cell-level or subcellular resolution and enable targeted detection of protein abundance and localization (Wu et al. 2024; Chen et al. 2025). However, in plant systems, their application is constrained by limited availability of validated antibodies, cell wall permeability barriers, and strong autofluorescence, which can reduce signal specificity and sensitivity.

MS-based SP provides a label-free alternative, typically using MALDI-based workflows combined with on-tissue digestion. These approaches enable detection of a broader range of proteins, but generally at lower spatial resolution and with reduced sensitivity compared with transcriptomic methods. Protein identification and quantification remain challenging due to complex fragmentation patterns and incomplete peptide coverage. Laser capture microdissection (LCM)-based spatial proteomics approaches can offer deeper proteomics profiling (thousands of proteins), but at the cost of extremely limited throughput (Liang et al. 2018; Xiang et al. 2023).

In practice, SP is particularly valuable when protein localization, post-translational regulation, or protein–protein interactions are central to the biological question, although its application in plant systems remains less developed than in animal models.

ST, SM, and SP occupy distinct positions within the experimental design space: ST provides broad, hypothesis-generating insight into gene expression programs, whereas SM and SP offer direct measurement of biochemical activity and functional state. The choice of platform should therefore be guided by the specific biological question, the required spatial resolution, and the availability of supporting resources such as reference atlases or validated reagents. Recently, studies have combined these approaches to leverage their complementary strengths (Ge et al. 2025; Yang et al. 2025).

### Quality control: resolution limits, molecular crowding, and more

Many of the technical constraints described above, including transcript diffusion, autofluorescence, molecular crowding, and ionization artefacts, cannot be completely eliminated experimentally. Quality control (QC) is therefore essential for distinguishing biological signals from technical bias. While studies and reviews in animal and human systems have begun to systematically address platform-specific biases and establish benchmarking frameworks for cross-platform comparison (Marx 2021; Ren et al. 2025; Grases and Porta-Pardo 2026), the practical consequences of technical artefacts remain insufficiently quantified and addressed within plant biology. A central challenge is that QC constraints are platform specific but biologically amplified in plant systems, due to cell size variability, and cell wall autofluorescence. As a result, both imaging- and sequencing-based approaches exhibit distinct, but complementary, sources of bias.

#### Sequencing-based methods: transcript accessibility, diffusion, and capture efficiency

Sequencing-based ST platforms face QC constraints centered on RNA accessibility and spatial fidelity of capture. Efficient transcript capture requires sufficient permeabilization to release RNA from plant cells, yet excessive permeabilization promotes lateral diffusion, redistributing transcripts beyond their original cellular boundaries (Giacomello and Lundeberg 2018). This trade-off is particularly acute in plant tissues, where rigid cell walls, variable lignification, and cuticular barriers create highly heterogeneous permeability landscapes across cell types and tissue regions.

Additional variability arises from capture surface interactions and tissue–slide contact, which can differ across sections due to mechanical distortion or embedding artefacts (Section 3.1). In high-water-content tissues, partial detachment or uneven compression can further exacerbate spatial inconsistencies (Giacomello and Lundeberg 2018), leading to spatially variable RNA capture that is not biologically meaningful. Together, these effects constrain the effective capture resolution. As a result, apparent spatial differences in transcript abundance may reflect technical variation rather than underlying biology. Metrics such as transcripts per unit area or per cell, spatial gradients in total RNA capture, and cell type–specific detection rates can be used to identify regions of under- or over-capture, across and within sections. Importantly, these measurements enable normalization strategies that account for local capture efficiency, allowing more robust comparisons across sections, conditions, and cell types, rather than overinterpreting technical artefacts as biological differences.

Several practical strategies can mitigate these constraints. First, systematic optimization of permeabilization conditions—for example, enzyme concentration, incubation time, and temperature—should be performed using tissue-specific pilot experiments, ideally guided by spatial QC readouts rather than global RNA yield alone (Giacomello and Lundeberg 2018). Second, recent work highlights the importance of spatially aware QC frameworks that explicitly account for local tissue context. For example, *SpotSweeper* (Totty et al. 2025) evaluates each capture location relative to its spatial neighborhood rather than applying global thresholds, thereby distinguishing true low-quality spots from biologically valid regions with intrinsically low transcript abundance. This approach reduces bias introduced by tissue heterogeneity and enables more consistent identification of compromised regions across spatial domains. Notably, such frameworks can also detect region-level artefacts, including areas of incomplete permeabilization (“dry spots”) or tissue damage, which may not be identifiable using conventional QC metrics but can substantially distort downstream analyses. Finally, QC-informed interpretation should be explicitly incorporated into downstream analyses. Rather than treating all spatial locations equivalently, analyses should account for local detection efficiency and effective resolution, particularly when comparing cell types with distinct structural properties or across developmental stages where tissue permeability may differ.

#### Imaging-based methods: optical resolution, molecular crowding, and probe specificity

High transcript density within confined cellular volumes can lead to molecular crowding, where either individual RNA molecules cannot be optically resolved or the assembly of complex sandwich-probes for signal amplification is hampered by sterically interference. This results in signal overlap, undercounting, and spatial blurring of transcript localization in imaging-based ST. In parallel, autofluorescence (Donaldson 2020) from cell walls, chloroplasts, and secondary metabolites reduces signal-to-noise ratios and can introduce spatially structured background signals. Together, these constraints define an effective optical resolution (Eng et al. 2019), which might be lower than the expected resolution of the imaging system. To address these challenges, QC must explicitly quantify background structure. Metrics such as spot density per unit area and local clustering of transcripts can be used to assess crowding and resolution limits. Similarly, measurement of background fluorescence intensity across channels and tissue regions provides a baseline for evaluating signal specificity.

Several complementary strategies can mitigate these limitations. First, probe design and gene selection are critical determinants of signal quality (Xia et al. 2019; Kuemmerle et al. 2024). Autofluorescence can be partially reduced through tissue clearing, chemical quenching, or photobleaching approaches prior to imaging. However, substantial residual autofluorescence often persists in specific plant cell types and structures (Donaldson 2020). In these contexts, optimization of probe sequences and fluorophore assignments to avoid spectral overlap with autofluorescence can substantially improve detection sensitivity. For example, excluding or minimizing the use of emission spectra that overlaps with strong cell wall or chlorophyll autofluorescence can reduce background interference and improve effective dynamic range (Jhu et al. 2026a). Second, inclusion of control probe panels—such as negative control probes targeting non-existent sequences or scrambled barcodes—provides an empirical estimate of background signal (Moffitt et al. 2016; Marco Salas et al. 2025). These controls can be used to define detection thresholds, enabling filtering of signals that fall within the range of nonspecific binding or autofluorescence-derived noise. This approach is analogous to traditional in situ hybridization workflows, where background staining defines the baseline for interpreting true signals. Finally, computational post-processing plays an essential role in QC and signal refinement. Background subtraction, spot deconvolution, and spatial filtering methods can improve signal discrimination, particularly when informed by experimentally derived noise distributions. However, as with sequencing-based correction, these approaches must be applied cautiously to avoid introducing artificial spatial structure.

#### Spatial metabolomics: ionization efficiency, annotation confidence, and spatial fidelity

SM introduces a distinct set of QC challenges because the measured signal depends not only on the abundance of a metabolite but also on ionization efficiency, local chemical environment, and confidence in metabolite annotation. For all MS-based workflows, ionization efficiency is a major consideration, as some molecules more readily ionize in positive or negative mode, and some molecules may remain neutral. As such, chemical derivatization approaches can be used to assist in ionizing the molecular classes of interest (eg phytohormones) while suppressing molecular classes that readily ionize (eg phospholipids), and these approaches have become more common in SM applications (Zemaitis et al. 2023).

In MALDI-based workflows, matrix deposition is a critical source of technical variability, as non-uniform matrix coverage can produce spatially structured differences in signal intensity that do not reflect underlying metabolite abundance. Excess solvent exposure during matrix application may also induce analyte delocalization, reducing spatial fidelity. Solvent-free application approaches, using sublimation, have aimed to address this issue, but analyte extraction from the sample into the matrix crystals can limit sensitivity (Spears et al. 2025). Moreover, ion suppression and matrix effects can vary across tissue regions with different chemical compositions, causing apparent differences in metabolite intensity that arise from local ionization conditions rather than biology. These issues are particularly relevant in plants, where chemically heterogeneous tissues, abundant secondary metabolites, and variable water content can strongly influence local ionization behavior.

Accordingly, QC for SM should include assessment of matrix uniformity (via high resolution optical or electron microscopy), mass spectral peak intensity of background signals, signal stability across the tissue, and the reproducibility of spatial patterns across replicate sections. Internal standards or reference compounds can be used to monitor technical variability and aid signal normalization. In untargeted metabolomics, many mass-to-charge ratio (*m/z*) features cannot be assigned unambiguously to a single compound. Biological interpretation should therefore avoid overinterpretation of spatial patterns based solely on nominal mass matching. Orthogonal measurements of detected analytes using pre-mass ion mobility or MSI with tandem MS are needed to improve confidence in molecular annotations (Veličković et al. 2018b; Zeng et al. 2025). Overall, the effective spatial resolution of SM is therefore not only defined by pixel size but also by analyte delocalization, annotation confidence, and local ionization behavior.

#### Spatial proteomics: antibody performance, epitope accessibility, and signal specificity

SP presents challenges that are strongly dependent on the underlying detection platform. In antibody-based approaches, signal quality depends on antibody specificity, epitope accessibility, and the consistency of staining, imaging, and background subtraction. In plant tissues, SP is further complicated by cell wall barriers and autofluorescence, but also variable fixation and antigen retrieval efficiency. All of these can produce spatially structured backgrounds or false-negative regions. As such, QC should include validation of antibody performance, assessment of background signal and spectral overlap, and evaluation of staining reproducibility across sections and experiments. In MS-based SP, analogous issues to SM arise, including non-uniform ionization, background peaks, and uncertainty in analyte identification.

Taken together, QC in spatial omics is not a secondary filtering step but a central determinant of biological interpretability. Without explicit QC, technical artefacts can be misinterpreted as biological patterns. A key emerging principle is that QC must be spatially aware, accounting for local tissue context rather than relying on global thresholds. Development of standardized and accessible QC metrics, like the benchmarking and best-practice frameworks established in the single-cell field (Grones et al. 2024; Fu et al. 2025), will be essential not only for robust data interpretation but also for enabling cross-study comparison, data reuse, and integration across platforms and species.

### Cell segmentation and spatial annotation in plant tissues

Accurate cell segmentation remains a major bottleneck in spatial omics and is particularly challenging in plant systems due to large intercellular spaces, multinucleated cells, and pronounced size heterogeneity across tissues. These features, together with signal attenuation in thick sections or deep Z-stacks, complicate the delineation of biologically meaningful cell boundaries and limit the robustness of automated approaches. Accurate segmentation is critical for reliable cell annotation, as it ensures correct assignment of transcripts to individual cells, which in turn determines cell identity and classification.

#### Image-based segmentation

Image-based methods rely on auxiliary structural signals, such as nuclear or cell wall staining, and are often implemented using deep learning frameworks including Cellpose (Stringer et al. 2021), Cellpose3 (Stringer and Pachitariu 2025), Cellpose-SAM (Pachitariu et al. 2025), PlantSeg (Wolny et al. 2020), CellSAM (Marks et al. 2025), MorphoGraphX 2.0 (Strauss et al. 2022), and StarDist (Weigert and Schmidt 2022). These approaches can capture biologically realistic cell morphologies when high-quality imaging is available, but their performance depends strongly on image quality and low noise levels for the accurate detection of cellular structures.

In plant tissues, nucleus-based segmentation can be particularly effective for densely packed cell types with relatively large nuclei, such as meristematic cells. However, segmentation becomes more challenging in large or highly differentiated cells, where nuclei are frequently displaced toward the cell periphery by large vacuoles or may be absent from thin tissue sections altogether, leading to under-segmentation, where multiple cells are erroneously merged into a single segmented object, or other segmentation errors. In contrast, cell wall staining often provides well-defined structural boundaries and can substantially improve segmentation accuracy in plant tissues. Additional staining workflows may not be equally compatible across platforms, fixation conditions, or tissue types, particularly in highly heterogeneous tissues. Consequently, incompletely resolved cell wall signals can still lead to under-segmentation and other segmentation artefacts. Successful applications of wall-based segmentation have nevertheless been demonstrated in plants. For example, Yao et al. (2024) used Fluorescent Brightener (calcofluor white) staining to label rice embryo cell walls and combined with Cellpose-based segmentation, achieved accurate cell boundary delineation while excluding RNA signals from intercellular spaces (Yao et al. 2024).

#### Transcript-based segmentation

Transcript-based approaches infer cell boundaries directly from the spatial distribution of RNA molecules, without requiring additional imaging channels. Methods such as Baysor utilize transcript density and identity to assign molecules to cells (Petukhov et al. 2022). More recently, ComSeg has been able to directly infer cell boundaries from single-molecule RNA coordinates without relying on membrane images or predefined cell-shape priors, making it well suited for complex tissues with irregular cell morphologies (Defard et al. 2024). While these strategies are well suited to imaging-based ST, they typically require nuclear signal, or assume nuclear position, and relatively uniform transcript distributions within cells, which may not hold in cases of strong subcellular localization, displacement by large central vacuoles, or polarized gene expression as recently observed in plant cells (Tennant et al. 2025), potentially leading to boundary inaccuracies.

#### Model-based and hybrid approaches

More recent methods aim to incorporate biologically informed constraints into segmentation. For example, Proseg formulates segmentation as a probabilistic inference problem inspired by physical simulations, allowing cell boundaries to emerge through iterative optimization while constraining unrealistic morphologies (Jones 2025). STCellBin, facilitates cell segmentation by leveraging models trained on ground-truth annotations derived from *Arabidopsis* seed cells (Zhang et al. 2024). In parallel, hybrid approaches integrate transcriptomic and morphological information to improve boundary delineation. For instance, RNA2seg is a deep-learning segmentation model that integrates RNA point clouds and membrane and nuclear staining data (Defard et al. 2026). These strategies represent an important conceptual advance, but their performance depends on model assumptions and data quality, and their general applicability across complex tissues requires further evaluation.

#### Context dependency and downstream impact

A key insight emerging through recent studies is that no single segmentation method performs robustly across all tissues, platforms, or biological questions (Yu et al., 2025). Mitchel et al. (2026) demonstrate that segmentation errors in imaging-based ST can substantially distort downstream analyses and propose local molecular neighborhood factorization as an effective strategy to detect and mitigate these errors (Mitchel et al., 2026). In addition, transcript-based segmentation accuracy is highly context-dependent and sensitive to parameter tuning, marker gene selection, and input data quality (Yu et al., 2025). Importantly, segmentation is not merely a preprocessing step: errors propagate directly into downstream analyses, fundamentally influencing cell annotation, gene expression quantification, inferred spatial relationships, and gene imputation. Subtle segmentation inaccuracies can potentially lead to altered clustering structure and misinterpretation of cell states, underscoring the need for careful segmentation curation and validation.

However, the applicability of cell segmentation depends on the underlying measurement modality and spatial resolution. While segmentation is central to imaging-based ST, where individual RNA molecules can be assigned to cells, this assumption does not hold universally across spatial omics. In some sequence-based ST and SM, signals may be acquired, depending on the methodology, at spot or pixel resolutions that can encompass multiple cells, limiting the feasibility of accurate cell segmentation. In these cases, alternative strategies such as spatial deconvolution or region-based clustering can be used to infer cellular composition or define spatial domains, rather than explicitly delineating individual cells.

#### Toward integrative pipelines in plant spatial omics

These findings shift the perspective from segmentation as a standalone computational task to a broader pipeline problem, in which experimental design, algorithm choice, and post hoc validation are tightly coupled. Integrative strategies that combine transcriptomic and morphological features can improve robustness, although their effectiveness is method-dependent, and manual quality control remains essential for validating automated predictions. Despite rapid methodological progress, systematic benchmarking of segmentation approaches in plant spatial omics is still lacking. Addressing this gap will require the development of plant-aware models that incorporate cell wall geometry and tissue-specific constraints, alongside standardized evaluation frameworks and improved multi-modal integration.

### Data integration: from spatial to single-cell and multi-omics

Data integration in spatial omics combines datasets across modalities or resolutions to enable label transfer, imputation, and cross-modal inference. Data integration between scRNA-seq and ST offers a great opportunity for in-depth analyses of different tissues without having to perform many cost- and labor-intensive additional experiments. Cell types identified in the scRNA-seq experiment can be mapped into a spatial context, and gene expression information for genes not measured in the ST experiment can be computationally imputed. However, differences in resolution, transcript coverage, and noise profiles complicate integration, particularly in plants where single-nucleus RNA sequencing data are often used as proxies for whole-cell expression (Farmer et al. 2021; Guillotin et al. 2023).

Tools such as Seurat v5 (Hao et al. 2024), Squidpy (Palla et al. 2022) and Scanpy (Virshup et al. 2023) provide flexible frameworks for data integration, although parameter choices can strongly influence results. Best practices for plants—such as how many anchor genes are sufficient, how to handle spatially restricted rare cell types, and how to assess transfer confidence—are not yet standardized.

Establishing universal guidelines is challenging because optimal parameters depend on the nature of the biological sample (eg a simple sample containing few distinct cell types vs. a complex sample containing many distinct cell types), and dataset characteristics, such as the number of cells within the sample, or the number of variable genes. Although bioinformatic tools often provide default values, their impact on the analysis is not always clearly communicated, and fixed defaults may not generalize across datasets. In some cases, parameters scaled to dataset size or complexity may therefore be more appropriate.

Data integration approaches are not limited to scRNA-seq and ST but already include approaches to integrate data across different omics technologies. Here, we will outline the challenges, limitations, and solutions relevant for integrating transcriptomics data, as well as data across technologies.

#### Integrating different spatial transcriptomics datasets

Integrating ST datasets across platforms, such as Visium and MERFISH, is complicated by differences in transcriptome coverage (whole-transcriptome coverage vs. targeted gene panel), sensitivity, and spatial resolution (multi-cell spots vs. single cells vs. subcellular resolution). Methods to align these data include batch correction and cell alignment using shared genes within both datasets to identify anchor cells which form the basis of integration (canonical correlation analysis or reciprocal primary component analysis with Seurat) (Hao et al. 2024), mutual nearest neighbors (MNN) approaches (Haghverdi et al. 2018), and graph matching with Conos or CAMEX (Barkas et al. 2019; Guo et al. 2026). Data pre-processing and careful consideration of the correct method still need to be applied to ensure high-quality integration data. Additional guidance for the researcher is often lacking, especially for the correct normalization of the input data, as well as the selection of comparison parameters (eg the selection of a suitable distance metric to determine cell similarity/dissimilarity).

#### Integrating spatial transcriptomics and single-cell data: gene expression imputation

Integration is often the preceding step to data imputation, the computational prediction of unmeasured gene expression in an experiment. However, this can be an invaluable tool for in-depth analyses of a specific tissue, while staying more labor- and cost-efficient.

Many imputation methods rely on aligning datasets based on a set of shared features first and then transferring expression using similar cells (cells with similar expression patterns, and/or close spatial neighbors) (Welch et al. 2019; Demesa-Arevalo et al. 2026). These methods are highly reliant on the shared genes between datasets, which influence data integration and, as such, the downstream data imputation. Using suitable distance metrics, carefully selecting genes to be used as imputation features, as well as accounting for datatype differences between datasets can increase the accuracy of these methods. Additionally, imputation accuracy is highly dependent on the quality of the cell segmentation of spatial data. Meanwhile, the reference dataset that is used to derive gene expression needs to contain as many cells as possible and cover all expected cell types/states to impute gene expression for a sparser dataset.

Other methods such as gimVI (Lopez et al. 2019) rely on probabilistic models but are often limited to datasets with larger gene panels. spRefine does not require a reference dataset (Liu et al. 2025) but rather relies on a pre-trained genomic language model, which might not always be available, particularly in plant sciences.

It is important to note that gene expression imputation is still limited by domain shifts between different technologies, and the number and identity (eg marker gene vs. housekeeping gene) of shared features and cell types and states across dataset. Additionally, using a gene expression imputation method often leads to smoothed gene expression profiles compared with the more heterogeneous, noisier gene expression profiles obtained through direct experimental measurements. Since the imputed gene expression patterns represent averaged or predicted values, any interpretation of the results and additional downstream analyses must be performed with caution and is still significantly strengthened by experimental validation.

#### Integrating spatial transcriptomics with other omics datasets

Several approaches already exist for integrating ST data with other omics data, such as proteomics, epigenomics, and phenomics. MARIO (Zhu et al. 2023) integrates scRNA-seq data (CITE-seq data) with single-cell proteomics data (CODEX data). Other approaches include Tangram for mapping paired single-cell RNA-seq and ATAC-seq data onto spatial data. Several tools were specifically developed for the integration of ST data with ATAC-seq data, such as the deep learning tool SpaDDM, based on directional diffusion models (Wang et al. 2026b); MultiGATE, which relies on the cells' interactions with neighboring cells and uses a graph attention autoencoder to learn a low-dimensional joint embedding (Miao et al. 2025); or spaMultiVAE, which is based on a variational autoencoder using a hybrid embedding consisting of 2 priors, which in turn can take spatial expression gradients into account (Tian et al. 2024). However, integration performance can depend strongly on the similarity between datasets and may decrease when cell populations or molecular features are poorly represented or absent in one of the datasets.

#### Integrating spatial metabolomics with other omics datasets

Unlike transcriptomic data, which are discrete and gene-annotated, metabolomic and proteomic datasets are largely continuous, chemically heterogeneous, and may lack unambiguous molecular identification. As a result, multimodal integration requires not only alignment of spatial coordinates but also reconciliation of fundamentally different data types, detection biases, and effective resolutions. Misaligned assumptions about spatial resolution or molecular diffusion can lead to overinterpretation if left unaddressed. In particular, standardization and guidance are needed for the pre-processing of the data to ensure good integration results and reproducibility.

Integration of SM with transcriptomic data has commonly relied on indirect association with single-cell or single-nucleus RNA-seq datasets, linking spatial metabolite distributions to cell identities and gene expression programs. For example, studies in *Taxus* have combined MALDI-MS with single cell transcriptomics to assign secondary metabolite biosynthetic pathways to specific cell types and identify candidate regulatory genes controlling taxoid production (Zhan et al. 2023). Similarly, work in *Panax* has integrated spatial metabolite distributions with single-cell transcriptomic trajectories to link metabolite accumulation to developmental states and tissue-specific biosynthetic activity (Yang et al. 2025). In these approaches, integration was achieved through spatial co-localization and correlation of metabolite intensities with the expression of known biosynthetic genes or pathway markers. While powerful, this strategy relies on indirect inference, as metabolites and transcripts are not measured within the same cells, and is therefore sensitive to differences in spatial resolution and sampling.

A complementary strategy integrates SM with ST data, allowing both modalities to be interpreted within a shared spatial framework. This can be achieved by aligning metabolite images with ST maps derived from adjacent sections or coarsened to comparable spatial resolution. Recent studies in cotton have combined ST, snRNA-seq, and SM to investigate fiber development and somatic embryogenesis (Ge et al. 2025; Sun et al. 2025). In these workflows, ST data provide the tissue context, while metabolite distributions are overlaid at matched or harmonized resolution, enabling the association of metabolic patterns with spatial gene expression domains. However, due to differences in spatial resolution and sample preparation requirements, such integration typically requires binning of transcriptomic data or interpolation of metabolite signals, which may reduce cellular resolution and introduce uncertainty in cross-modal alignment.

Recent advances in animal models have begun to enable more direct integration of SM and ST. These include workflows that allow sequential acquisition of metabolomic and transcriptomic data from the same tissue section, as well as computational frameworks for spatial co-registration and multimodal data integration (Ravi et al. 2022; Liu et al., 2026b). Such approaches reduce reliance on indirect interference and improve alignment between modalities, highlighting potential directions for future development in plant spatial omics.

Data acquired from SM have also been employed to inform SP measurements of the same sample. Explicitly, the metabolome-informed proteome imaging workflow utilizes MSI-based SM data to determine where to acquire LCM-based SP measurements (Veličković et al. 2024, 2025). LCM liquid chromatography tandem MS measurements can provide deep untargeted proteomics information of specific regions of a sample (thousands of proteins from cell clusters of a couple of hundred cells) (Liang et al. 2018). However, this SP approach is very data rich, but it is extremely slow (∼1 pixel/h). As such, using SM findings can identify which areas to micro-dissect for deep proteomics. This approach can be performed on serial sections or the same tissue section sequentially.

To summarize, there are different approaches for integrating data across different modalities and omics technologies, which mainly rely on shared latent representations, graph-based methods, and deep generative frameworks to overcome the differences of these diverse types of datasets. However, many challenges remain, such as modality-specific biases, strong dependency on shared features or cell populations across datasets, resolution mismatches, and the limited availability of large-scale experimentally validated ground truth data.

### Spatial data analysis

By incorporating physical coordinates, spatial analyses can identify tissue domains, molecular gradients, and cellular neighborhoods that are not directly recoverable from dissociation-based datasets (Liu et al. 2023b; Moses et al. 2023; Walker and Nie 2023; Zormpas et al. 2023; Klein et al. 2025; Peirats-Llobet et al. 2026). One core analysis is the identification of spatially variable genes (SVGs), whose expression differs significantly across tissue locations. SVGs can reveal how tissue organization, cellular interactions, and regional specialization are encoded at the molecular level and may highlight genes involved in signaling, transport, or developmental regulation. Tools like Squidpy (Palla et al. 2022) and Spateo (Qiu et al. 2024) include dedicated modules for SVG detection. Similar concepts can be extended to other modalities, including SM and SP, where spatially variable metabolites or proteins can provide direct insight into biochemical activity and functional states.

Spatial datasets also enable “niche analysis”, which focuses on identifying cellular microenvironments (niches) defined by specific combinations of neighboring cell types. In plants, changes in local niche composition may alter differentiation trajectories during organogenesis or modulate cellular responses under biotic or abiotic stress. Tools such as CellCharter (Varrone et al. 2024) and NicheCompass (Birk et al. 2025) support the identification and characterization of spatial cell niches.

Spatial omics also provides an opportunity to study cell–cell communication directly in tissue context. Conceptually, niche analysis emphasizes the organization of cellular communities and their local microenvironments, whereas communication analysis focuses more explicitly on the molecular signals exchanged between cells, although recent methods increasingly integrate these 2 levels. In plants, intercellular communication classically involves plasmodesmata, but secreted peptides have emerged as key regulators of spatially precise developmental signaling (Zhong et al. 2022). A growing number of plant-specific ligand–receptor resources and computational frameworks, such as PlantPhoneDB (Xu et al. 2022) and PlantCellChat (Liu et al. 2026a), are beginning to enable such analyses in plant single-cell and spatial datasets.

Beyond descriptive mapping, spatial analyses can support spatial gene regulatory network inference (Littman et al. 2023) to help generate and test hypotheses about cell-autonomous vs non–cell-autonomous responses and facilitate the testing of mechanistic models of pattern formation (Almet et al. 2024; Weissbart and Formosa-Jordan 2026).

Developing spatially aware computational models of growth, differentiation, and signaling remains an open challenge but represents a major opportunity for conceptual advances. Such spatial models will likely rely on a small number of variables to study specific developmental questions. These types of minimal, low-dimensional models reflect the vision from Waddington landscape representing the developmental path of a system being canalized in a low-dimensional space (Waddington 1957; Saez et al. 2022). Bridging connections between such low-dimensional models and the high-dimensional datasets derived from spatial omics represents a major challenge. Although there are several existing dimensionality reduction techniques, not all of them are appropriate for establishing connections with these minimal models (Maehara and Ohkawa 2025). Recent dimensionality reduction techniques have been shown to preserve key geometrical information of the scRNA-seq data, making them more appropriate for minimal models (Schiebinger 2021; Cislo et al. 2025; Fontaine et al. 2025; Maehara and Ohkawa 2025). In the future, it will be important to promote the development and application of novel dimensionality reduction approaches that preserve key information and extend them in space.

### Moving to 3D

2D sections provide an inherently incomplete view of plant tissues, where cellular organization and connectivity, developmental trajectories, and interactions with the environment extend across all 3 spatial axes. While current spatial omics platforms have enabled unprecedented molecular resolution in individual sections, reconstructing the true 3D architecture of plant organs remains a central challenge. Early efforts to extend spatial omics into 3D relied on integration of serial sections, exemplified by packages such as STUtility and Semla (Bergenstråhle et al. 2020; Larsson et al. 2023). These approaches are well suited to plant systems, where tissue geometry and developmental progression are often predictable. STUtility enables alignment, joint normalization, clustering, and visualization of consecutive Visium sections, allowing gene expression patterns to be examined across tissue depth. Although STUtility does not explicitly reconstruct true 3D coordinates, it captures continuity across serial sections and effectively supports “2.5D” analysis (Peirats-Llobet et al. 2026; Wu et al. 2026).

Building on these serial reconstruction strategies, recent advances suggest that approaches to 3D spatial omics can be broadly categorized into several emerging paradigms that increasingly leverage machine-learning–driven integration. One strategy combines high-resolution spatial profiling with computational alignment of serial sections to generate volumetric reconstructions. Sequencing-based platforms (eg Open-ST and others) exemplify this approach, enabling near single-cell resolution and integration with histological imaging (Schott et al. 2024). SCiLS can enable 3D reconstruction of MSI, enabling SM finding of metabolite asymmetry across an entire plant organ (Veličković et al. 2018a). However, these strategies remain sensitive to sectioning artefacts, tissue deformation, and alignment errors that can distort cellular continuity across slices (Yan et al. 2026).

A second approach aims to reconstruct 3D tissue organization through computational inference, directly integrating molecular similarity with spatial information across multiple sections. Methods such as STAIR use graph-based embeddings and attention mechanisms to jointly align slices and infer their relative positions along the z-axis, improving scalability and enabling cross-platform integration (Yu and Xie 2025). However, as highlighted by recent benchmarking studies, these approaches rely heavily on the joint use of transcriptional similarity and spatial proximity and may lack sufficient physical constraints, particularly in heterogeneous or developmentally dynamic tissues (Hu et al. 2024; Yan et al. 2026).

Third, a conceptually distinct strategy is emerging through direct volumetric profiling, in which ST measurements are acquired from intact thick tissue blocks rather than reconstructed from sections. Techniques such as Deep-STARmap enable in situ sequencing within 60- to 200-μm tissue volumes, preserving native cellular context and eliminating the need for post hoc alignment (Gandin et al. 2025; Sui et al. 2025). These approaches provide true 3D molecular information and enable simultaneous analysis of transcriptional and translational states but currently rely on targeted gene panels and remain technically demanding, limiting their immediate implementation in plant systems.

Fourth, a plant-relevant strategy is emerging through whole-mount ST, which enables gene expression profiling directly in intact tissues without physical sectioning. Methods such as PHYTOMap (Nobori et al. 2023) combine padlock probe-based detection, rolling-circle amplification, and sequential hybridization to achieve multiplexed whole-mount spatial profiling at single-cell resolution in intact tissues (Ke et al. 2013), although a limited number of transcripts can be investigated. By avoiding physical sectioning and computational reconstruction, PHYTOMap minimizes spatial distortion and preserves biologically relevant context, including spatial relationships between plant cells and associated microbial partners. These advantages come with practical constraints. Achieving sufficient probe penetration through intact plant cell walls and cytoplasm requires multi-step enzymatic pretreatment (eg cell wall–digesting enzymes and proteinase K). Current implementations perform best in small, optically accessible tissues such as Arabidopsis root tips, where geometry and cell wall properties are favorable (Nobori et al. 2023). However, permeability varies across plant species, tissue types, and developmental stages, necessitating context-specific optimization. Imaging depth is further limited by light scattering and cell wall autofluorescence, restricting robust 3D analysis unless optical clearing approaches (eg ClearSee) (Kurihara et al. 2015) are incorporated. Multiplexing capacity is subject to similar trade-offs. Current implementations typically profile tens of genes per experiment, with higher multiplexing achievable through additional hybridization rounds at the cost of increased imaging time and cumulative signal degradation.

Emerging strategies aim to extend these limits by enabling more targets to be detected within a single fluorescence channel. Intensity-based approaches such as PRISM distinguish targets using discrete fluorescence intensity levels rather than separate spectral channels, enabling multiplexing within a single imaging round without additional fluorophores or hybridization cycles (Chang et al. 2025). In parallel, combining whole-mount hybridization-based ST with microbial labeling approaches—including fluorescent reporter strains or rRNA-targeted fluorescence in situ hybridization (FISH) and its highly multiplexed extensions (eg HiPR-FISH [Shi et al. 2020])—offers a route to visualizing plant–microbe interactions at single-cell resolution in intact tissue. Extending this to strain-level resolution within complex, multi-member communities would enable spatially resolved analysis of host–microbiome organization that is not currently achievable and remains an important methodological challenge. The transgene-free nature of PHYTOMap facilitates integration with complementary readouts, including genetically encoded reporters for hormones (Brunoud et al. 2012; Rowe et al. 2025; Tang et al. 2025), nutrients (Ho et al. 2009), or metabolites (De Michele et al. 2013; Dundas et al. 2026). PHYTOMap is therefore well suited for integrating spatial transcriptional, microbial, and reporter-based readouts within intact tissues.

Across all paradigms, a major bottleneck lies in 3D cell segmentation (Gandin et al. 2025; Sui et al. 2025) and annotation. Accurate segmentation is essential for defining cellular neighborhoods, quantifying cell–cell interactions and interpreting spatial gene expression in a biologically meaningful framework. In addition, 3D spatial omics generate extremely large and computationally intensive datasets, creating major challenges for image registration, volumetric alignment, and comparative analysis across biological conditions. These challenges also present an important opportunity to investigate how gene expression programs are coordinated with cell size, shape, position, and tissue architecture during development and environmental responses. Notably, the field of plant biology has already developed advanced 3D segmentation frameworks, such as MorphoGraphX (Barbier de Reuille et al. 2015; Strauss et al. 2022), that integrate high-resolution imaging with geometry-aware analysis to extract cell shape, growth, and lineage information. These approaches bridge 2D and full 3D representations, enabling quantitative, time-resolved analysis of tissue morphodynamics. Integrating such frameworks with ST presents a major opportunity to link cellular geometry, lineage, and molecular state, advancing toward a fully 4D understanding of plant development.

Rather than converging on a single dominant platform, plant 3D spatial omics will likely advance through integration of serial reconstruction, volumetric imaging, whole-mount profiling, and geometry-aware computational frameworks. The central challenge is no longer simply generating spatial maps but resolving how molecular state, cellular geometry, and developmental trajectories are coordinated across intact organs through time.

Low-multiplex whole-mount imaging methods, including smFISH, HCR RNA-FISH, and PHYTOMap, prioritize quantitative accuracy, 3D whole-mount compatibility, and practical accessibility over gene plex. Their major strength lies in providing quantitative single-molecule measurements in intact tissues using technical assumptions largely independent from sequencing-based and high-plex imaging approaches. Consequently, these methods increasingly function as reproducibility infrastructure for validating spatial patterns identified by Xenium, MERFISH, Stereo-seq, and related platforms. PHYTOMap is particularly attractive in plants because its independence on specialized commercial instrumentation, its compatibility with whole-mount tissues using standard confocal microscopy, and its low cost (Nobori et al. 2023). The primary bottleneck is therefore the lack of robust, transferable protocols across diverse plant tissues and species. As high-plex spatial technologies continue to scale, the importance of accessible orthogonal validation frameworks will likely increase rather than diminish (Fig. 2).

## Recommendations for the field

### Protocol sharing and shared resources

As plant spatial omics transitions from proof-of-concept studies to large-scale biological discovery, the lack of standardized and easily accessible protocols remains a major bottleneck. In contrast to animal systems, plant tissues pose distinct technical challenges that require highly specialized, often species- or organ-specific handling. The large variety of spatial platforms and workflows has made it increasingly difficult for plant researchers to keep pace with the best practice methodologies. These challenges not only complicate protocol transferability but also hinder reproducibility across laboratories (Valihrach et al. 2024). To address this, we advocate for a coordinated, community-driven effort toward open protocol sharing and resource development.

We recommend establishing an open-access repository for plant spatial omics protocols, ideally built on or integrated with existing platforms such as protocols.io (Teytelman et al. 2016), alongside protocol-focused journals, methodological book series, and community repositories that support detailed workflow reporting (Wilkinson et al. 2016; Grones et al. 2024). These resources should host detailed, version-controlled, and community-curated workflows covering all critical experimental stages. Importantly, such repositories should highlight platform- and tissue-specific adaptations, enabling the translation of widely used technologies across diverse plant species, organs, and developmental stages. Broader adoption of standardized and interoperable protocol-sharing frameworks, together with shared optimization datasets, troubleshooting records, and validated reference workflows, would substantially accelerate reproducibility, benchmarking, and method development across plant spatial omics.

For imaging-based ST approaches, the probe design is a crucial and often limiting factor for plant researchers attempting to apply the technology to their species (Plummer et al. 2026). We recommend creating species- and tissue-specific probe design repositories that include annotations of probe performance (specificity, sensitivity, cross-reactivity) and guidelines for selecting gene families and paralogs common in plant genomes. Accurate cell type identification and annotation is crucial for solving biological questions. While scRNA-seq has been widely applied in plant science studies and has identified numerous marker genes, these markers are not always robust in spatial contexts due to differences in detection sensitivity and spatial regulation. We therefore propose to develop a curated repository of spatially validated marker genes, encompassing diverse plant species, organs, and developmental stages. This resource should prioritize markers validated through orthogonal approaches such as in situ hybridization or high-resolution ST. Establishing a plant spatially validated cell type marker database, which includes features such as the expression patterns, tissue specificity, and cross-species conservation of marker genes, would significantly enhance the robustness of both single-cell and ST data analysis. Extending this concept beyond transcriptomics, curated databases of spatially resolved metabolites and protein markers would further enhance cross-modal integration and interpretation, particularly for linking gene expression patterns to biochemical and functional outputs.

To maximize the utility of shared protocols and datasets, we further recommend the adoption of minimum reporting standards for plant spatial omics experiments. These should include detailed metadata describing: (1) tissue growth conditions and developmental stage; (2) section thickness and orientation; (3) fixation and permeabilization conditions and timing; and (4) platform-specific parameters and sequencing depth or probe designs.

The importance of standardized metadata and data structures has been widely emphasized in spatial omics, as they enable reproducibility, benchmarking, and cross-study integration. Database efforts such as STOmicsDB explicitly incorporate structured metadata and standardized data archiving systems to ensure interoperability and reuse of spatial datasets (Xu et al. 2024). Similar standards, adapted to plant-specific experimental variables, would be essential for building a comprehensive and reusable data ecosystem. For SM and SP, metadata standards should additionally include parameters such as matrix composition and deposition method, ionization conditions, MS settings, and antibody panels or probe validation methods. Studies that employ MSI approaches should, at minimum, use the Self-Monitoring, Analysis, and Reporting Technology (SMART) data reporting standard (Xi et al. 2023).

Finally, we propose leveraging advances in AI (or large language models, LLMs) to facilitate the standardization and dissemination of plant spatial omics protocols and workflows and the derived analyses. New computer vision-based AI pipelines (Zhu et al. 2026) still need to be tested in plant systems and could help overcome the current limitations described here. An AI tool trained from pre-validated spatial analysis tools has been shown to be capable of improving the reproducibility and robustness of spatial analysis workflows (Yang et al., 2026). A domain-specialized AI system, tailored using curated collections of plant spatial omics protocol and benchmarking datasets, could enable context-aware suggestions for tissue preparation, probe design based on targeted species, tissues and platforms. Such systems could be particularly valuable for multimodal experiments, where optimal conditions for transcriptomics, metabolomics, and proteomics often conflict and require coordinated optimization (Chen et al. 2026).

### Toward standardized workflows and benchmarking in plants

A challenge in plant spatial omics is that community best practices have not yet been established. Without standardized workflows, researchers may experience extended periods of trial and error, often repeated independently across laboratories. This results in suboptimal outcomes and ultimately increases overall costs. We propose shared evaluation criteria and standard workflow practices specific to plants that can provide clarity and reproducibility as opposed to using resources designed for bacterial, fungal, human, and mouse cells (Ma et al. 2024). This workflow encompasses both the preprocessing and data analysis stages for spatial omics. Preprocessing data includes quality control, normalization, and orienting expression on a spatial matrix, whereas the analysis encompasses cell segmentation, dimensionality reduction, clustering, expression analysis, and visualization (Lee et al. 2025b).

Cell segmentation is critical for accurate cell-typing. Unlike human and mouse tissue that benefit from nuclear expansion segmentation techniques, the variety of sizes, shapes, nuclei count, and the presence of cell walls and large vacuoles bring additional factors to consider. Existing generalist models like Cellpose3 and Omnipose are commonly accepted for out-of-the-box segmentation, but they are not specialized to plants and do not accurately account for the specific characteristics of plant cells (Cutler et al. 2022; Vijayan et al. 2024; Stringer and Pachitariu 2025). Meanwhile, collaborative efforts toward generalist cell segmentation models have made strides to more accurately and efficiently segment multi-modal images, but unfortunately, these new models are not ideal for plants because they primarily used single-cell yeast and bacteria images for training (Cutler et al. 2022; Lee et al. 2022; Ma et al. 2024; Stringer and Pachitariu 2025). The plant spatial omics would greatly benefit from a similar collaborative effort to develop plant specific models. This effort would require a large, well-curated cache of multi-modal images for training purposes, including datasets with cell wall stains. Such a collaborative strategy would support the development of robust plant-specific models and could establish a community standard for future analyses.

Benchmarking efforts in plant spatial omics should also address modality-specific challenges in metabolomics and proteomics. For SM, this includes evaluating metabolite annotation confidence, ionization variability, and spatial fidelity under different sample preparation and acquisition conditions. For SP, benchmarking should focus on antibody specificity, reproducibility of staining, and consistency across multiplexed measurements. Establishing standard evaluation metrics for these modalities will be essential for ensuring data quality and enabling meaningful cross-study comparisons.

Beyond benchmarking segmentation approaches to spatial omics for plants, the field needs standardized best practices for determining the optimal numbers of biological and technical replicates, tissue sections, and cells. Finding the ideal balance of statistical significance in the experimental design and the resources used is of utmost importance for effective and efficient science (Wagner and Kleiner 2025). We propose the assembly of a community benchmarking consortium. The goals of this consortium would be: (1) initial collaborative benchmarking of cell segmentation and data analysis methods for plant omics; (2) best practice recommendations, including sample size, quality control metrics, noise reduction, integration, data storage, and data sharing; (3) curation of data preprocessing pipelines; and (4) a published journal article to address these issues. Members of this consortium would commit to attending consortium meetings, participating in discussions, sharing multi-modal plant images and datasets, and contributing domain-specific expertise for the resulting journal article. The consortium committee would be tasked with organizing the conference, guiding discussions, and coordinating members' contributions to the paper.

The increasing adoption of multimodal spatial omics highlights the need for coordinated standardization across modalities. Integrating ST with metabolomics and proteomics requires harmonization of sample preparation workflows, spatial resolution, and data structures. Community-driven efforts could play a big role in the development of shared experimental designs and benchmarking frameworks that explicitly address cross-modal integration, ensuring that multimodal datasets can be reliably combined and interpreted.

### Empowering broader adoption through practical guidance

The rapid expansion of spatial omics technology necessitates a structured approach to platform selection to lower entry barriers for plant researchers. The strength of spatial omics is that it provides the spatial context within the tissue, which is helpful for investigating cell heterogeneity and cell to cell communications. A researcher stepping into this field should first determine if the biological question requires spatial context (Fig. 3). If research focuses on a specific cell population (eg pollen) for which effective cell enrichment methods are available, or on differential expression at the tissue level rather than mapping cells to the microenvironment (Birnbaum et al. 2005; Washburn et al. 2023), then traditional assays or scRNA-seq may remain the most efficient and cost-effective approaches.

**Figure 3:**
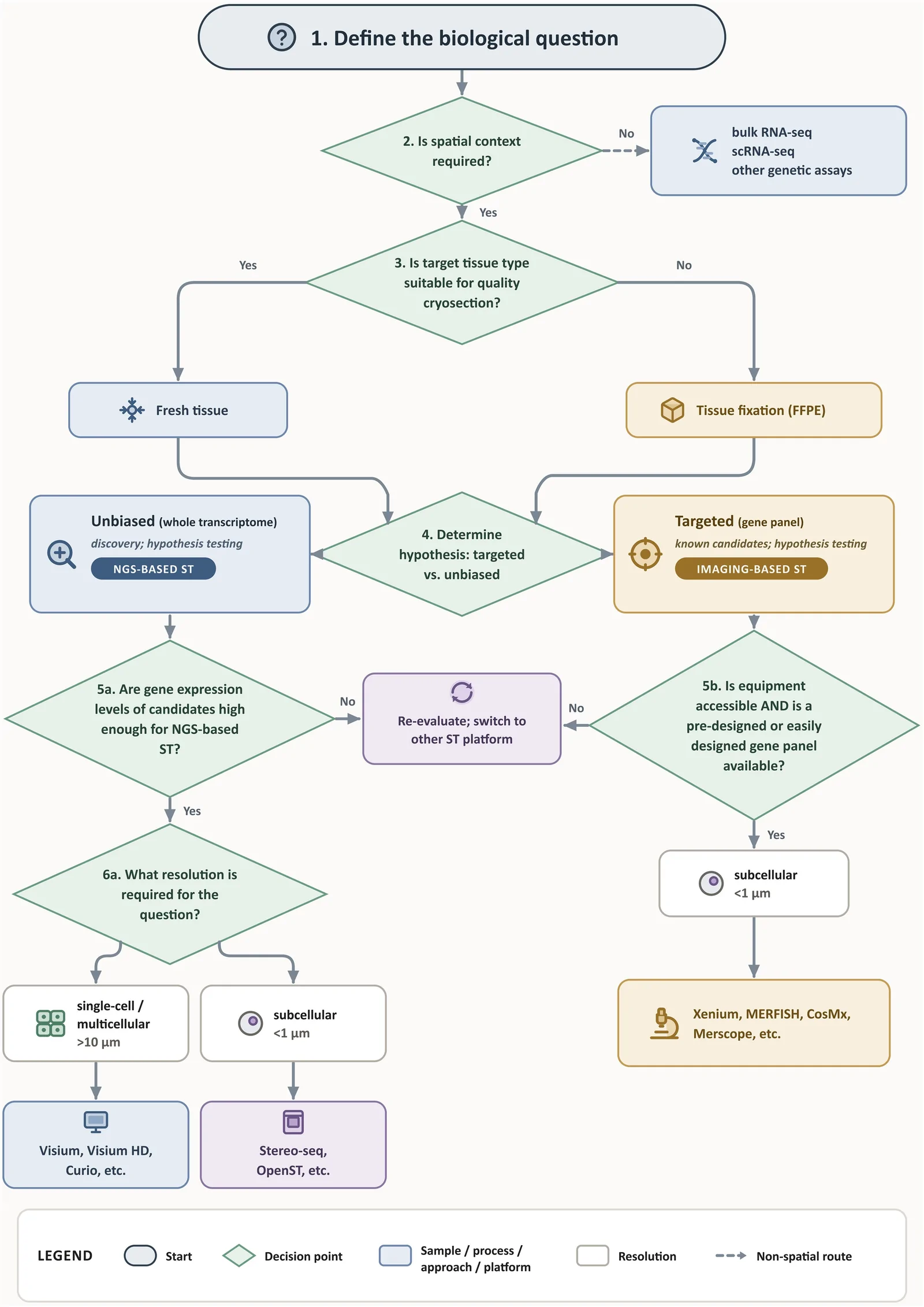
Decision framework for selecting ST approaches in plant tissues. The workflow summarizes key considerations for selecting an appropriate ST strategy, beginning with definition of the biological question and whether spatial context is required. Tissue suitability for cryosectioning guides the choice between fresh tissue and FFPE processing. Downstream platform selection is then determined by the experimental hypothesis, transcriptome coverage, candidate gene expression, equipment or panel availability, and required spatial resolution. Unbiased NGS-based ST approaches are suited for discovery or hypothesis testing, whereas targeted imaging-based platforms are appropriate for known gene panels and subcellular-resolution analyses.

If the spatial context is required, the next critical hurdle is the tissue suitability assessment, where researchers must evaluate if the target tissue can yield high-quality sections (Fig. 3). Plant-specific factors that were described previously, such as the presence of secondary metabolites and irregular surfaces, determine whether the project proceeds with fresh-frozen tissue for cryosectioning or moves toward FFPE protocols. Following this, researchers can determine the ST platforms based on the specific research goal: an unbiased, whole-transcriptome approach for discovery-based studies or a targeted gene panel for hypothesis testing. For discovery-based research, NGS-based ST is the standard route, provided that anticipated expression of candidate genes is high enough to meet the platform's detection sensitivity limits. Conversely, imaging-based platforms provide higher sensitivity for targeted genes but require a feasibility assessment: is a pre-designed or easily customizable gene panel available for species of interest? Final platform selection is then refined by the required resolution, ranging from multicellular or single-cell tools like Visium HD 3′ (2 μm), or Curio (10 μm) to subcellular or single-molecule resolution provided by Stereo-seq, Xenium, or MERFISH (<1 μm).

To help researchers adopt spatial omics, we propose using the shared protocols and dataset repositories mentioned earlier to create “researcher starter kits.” These kits would serve as a practical starting point, offering high-quality benchmark datasets and pre-configured quality control (QC) pipelines. By providing ready-to-use scripts (such as Jupyter Notebooks or R scripts) optimized for plant-specific issues, these resources help researchers avoid the struggle of building workflows from scratch. Furthermore, these kits allow users to validate their local computational environments against “gold standard” results before processing their unique biological samples. Providing this kind of hands-on guidance, alongside centralized repositories, is a key step toward making spatial omics accessible to the wider plant science community.

### Data sharing and reproducibility

Comprehensive data sharing should include raw and processed data, metadata, segmentation models, code, and spatial browsers. Without these components, reproducibility and reuse remain limited. The FAIR guiding principles (Wilkinson et al. 2016) outline the importance and criteria to make data Findable, Accessible, Interoperable and Reusable. During the last years, guidelines have been recommended regarding the reporting of single-cell RNA-seq experiments, minSCe, the Minimum Information about a Single-Cell Experiment (Füllgrabe et al. 2020), and the best practices for not only the execution, but also analysis of single-cell transcriptomics in plants (Grones et al. 2024). Both guidelines recommend relevant metadata to be recorded to improve overall dataset FAIRness, including information about the biological sample, sample preparation, sequencing libraries, sequencing results, raw data, processed data, validation, and data availability. However, ST poses additional challenges due to increased data diversity. Thus, raw experimental data, which includes the sequencing or image data, are critical for data reusability. To ensure interoperability and data sharing, open common formats are preferred over software-specific, proprietary formats. While an initial raw data processing step might be limited to specific proprietary software, providing the first open-format result or a data conversion will enable reusability and facilitate collaboration between different organizations that might not have access to the same software. Since more detailed analyses are often performed using bioinformatics solutions such as Seurat (for R) (Hao et al. 2024) or Scanpy (for Python) (Wolf et al. 2018; Virshup et al. 2023), data sharing is greatly enhanced by providing processed data such as the count matrices, coordinates, and additional gene- or cell-associated metadata in the respective data objects (eg Seurat objects in Seurat, Anndata in Python), but additional information regarding data object version must be provided (eg the Seurat object underwent major structural changes between v4 and v5).

In ST analyses, methods used for cell segmentation, spot deconvolution, and transcript assignment should be carefully documented, particularly when custom-trained or fine-tuned deep-learning models are employed. As segmentation and image-analysis tools continue to expand rapidly, clear annotation and reporting standards for these procedures are becoming increasingly important. Similarly, sharing complete analysis workflows, intermediate outputs, and executable code through repositories such as GitHub is becoming standard practice, with detailed tool and dependency versioning essential for full reproducibility.

Metadata information regarding the computational analysis in a common format has not yet been established. While guidelines for reporting standard scRNA-seq experiments already exist, extensions to ST are necessary to capture all relevant information in these more complex experiments. One demonstrated approach toward documenting a scRNA-seq/ST experiment was used in a study on barley (Dörpholz et al. 2025; Demesa-Arevalo et al. 2026), where the research data infrastructure from the German consortium DataPLANT was used (Weil et al. 2023). DataPLANT established a FAIR digital object called ARC (annotated research context), which combines the principles of the ISA standard (investigation—study—assay) (Sansone et al. 2016), CWL (Common Workflow Language) (Crusoe et al. 2022), and RO-Crate (research object crate) (Soiland-Reyes et al. 2022), to enable ontology-driven, machine-actionable (meta)data. Data are shared through the GitLab instance PLANTdataHUB, which facilitates communication and collaboration between project partners, while tracking the individual changes made by each participant. Within the barley study, this framework was used to describe experimental workflows from the lab as ISA sheets, including the plant growth procedure, the protoplasting, sample pooling, bulk and single-cell RNA sequencing, gene identification and annotation, and molecular cartography and cell segmentation with a web interface. Each ISA sheet starts with an input identifier for a sample and ends with an output identifier in each row, unambiguously linking all different sample preparation and assay results together. The subsequent analyses such as quality control, data integration, cell clustering, and gene expression imputation, which were performed using R and Python, were wrapped with CWL in combination with Docker containers, which contain all bioinformatics tools and dependencies in the correct versions that are needed to fully reproduce the results. The modular approach of splitting the analysis workflow into individual executable steps allows for high flexibility, allowing other researchers to reuse the entire workflow, or only individual steps.

To further improve accessibility and explorability of the data, multiple different web browsers have been set up in conjunction with specific ST experiments. Such browsers include the Arabidopsis Developmental Atlas Viewer, which is based on the CZ CELL × GENE viewer (Lee et al. 2025a), the STomicsDB Maize Development Ear Spatial Transcriptome Atlas (Wang et al. 2024), the WebAtlas Visualizer of Multiplexed Error-Robust Fluorescence In Situ Hybridization (MERFISH) dataset in wheat inflorescence tissue (Long et al. 2025), the barley virtual in situ transcriptome atlas BARVISTA (Demesa-Arevalo et al. 2026) or the barley grain spatial transcriptome atlas (Peirats-Llobet et al. 2026). While these browsers each serve a similar purpose, a unified browser for plants is currently missing, and other platforms, such as CZ CELL × GENE (Program et al. 2025), limit their datasets explicitly to human and mouse data. However, establishing guidelines and best practices for metadata within the plant ST community will be a first step towards providing more unified resources for researchers.

## Outlook: a call to action

Plant spatial omics have reached a pivotal moment. The field is moving beyond proof-of-concept demonstrations toward studies capable of resolving how cells, tissues, metabolites, proteins, and environmental signals are spatially coordinated during plant development and adaptation. However, this transition will depend not only on more advanced technologies, but also on the community's ability to address the practical bottlenecks that currently limit reproducibility, scalability, and accessibility.

A major opportunity now is to build shared infrastructure for plant spatial omics. Community-curated protocols, benchmark datasets, validated marker and probe resources, and plant-specific analysis standards would reduce duplicated optimization efforts and allow new users to enter the field more efficiently. Given the high cost and technical complexity of many spatial omics platforms, collaborative implementation models involving shared instrumentation, centralized processing pipelines, or multi-group sequencing consortia may further improve accessibility and experimental scalability, particularly for plant research communities with limited local infrastructure. Such resources would also help shift spatial omics from a specialist technology toward a routine experimental framework for plant biology. Importantly, these efforts should span transcriptomics, epigenomics, metabolomics, and proteomics, enabling multimodal spatial datasets that connect gene expression with biochemical and functional outputs.

Looking forward, the integration of spatial omics with artificial intelligence offers an especially promising direction. Large, well-annotated spatial datasets across species, tissues, developmental stages, and environmental conditions could support the development of plant-specific foundation models capable of learning conserved and divergent principles of tissue organization. These models could eventually assist with cell type annotation, cross-species comparison, spatial pattern prediction, experimental design, and multimodal data integration. However, their success will depend on high-quality community datasets, transparent metadata, reproducible workflows, and shared evaluation standards.

Ultimately, the next phase of plant spatial omics will require both methodological innovation and collective coordination. By sharing protocols, establishing benchmarking consortia, developing interoperable data resources, and embracing AI-enabled analysis frameworks, the plant science community can transform spatial omics into a robust, scalable, and broadly accessible approach. These efforts will not only improve technical reproducibility but also open new opportunities to understand how plant tissues are built, how they respond to changing environments, and how spatially resolved biology can inform crop improvement and sustainable agriculture.

## Data Availability

No new data were generated in this review article.

## Use of artificial intelligence

Writing support for preparation of this manuscript, including summarizing workshop notes and talk transcripts and improving readability, was provided with assistance from an AI language model (ChatGPT 5.5). Original figure drafts were created by the authors and subsequently refined with assistance from ChatGPT 5.5 and Claude Opus 5 for artistic improvement under careful author supervision and instruction. All content was comprehensively reviewed, edited, and approved by the authors, who take full responsibility for the final manuscript.
